# The Use of Sublaminar Wiring in the Sub-Axial Cervical Spine as an Adjuvant to Lateral Mass and Pedicle Screw Fixation in a Patient with Metastatic Carcinoma of the Upper Thoracic Spine

**DOI:** 10.7759/cureus.6671

**Published:** 2020-01-15

**Authors:** Calvin C Whaley, Michael Young, Jason M Seibly

**Affiliations:** 1 Neurosurgery, Advocate Health Care, Normal, USA; 2 Neurosurgery, Advocate Health Care, Oak Lawn, USA; 3 Neurosurgery, Central Illinois Neuroscience Foundation, Bloomington, USA

**Keywords:** sublaminar wire, spinal oncology, spinal instrumentation, cervico-thoracic fusion

## Abstract

A 60-year-old male presented with kyphotic deformity caused by a non-small cell lung cancer metastasis in the cervical-thoracic junction. His pathology caused spinal cord compression and segmental instability. The patient underwent a posterior decompression to try and improve neurological function as well as posterior lateral mass and pedicle screw fixation crossing the cervical-thoracic junction to stabilize his instability. A novel technique incorporating sublaminar wiring across a cross-link was utilized to increase pull out strength of the superior lateral mass screws. Also included is a discussion regarding the safe use of sublaminar wires, the history of posterior cervical and thoracic fusion, and the prevalence of instrumentation failure.

## Introduction

Multiple methods of posterior cervical and thoracic rigid fixation have been developed to create stability for the spinal column in situations of trauma, infection, and malignancy [1,2]. This article presents the case of a 60-year-old male with metastatic lung cancer, spinal canal compromise, and a notable kyphotic deformity from malignant invasion of the left half of his upper thoracic vertebra as well as a novel method of wiring to attempt to reduce lateral mass screw pull out.

## Case presentation

**Figure 1:**
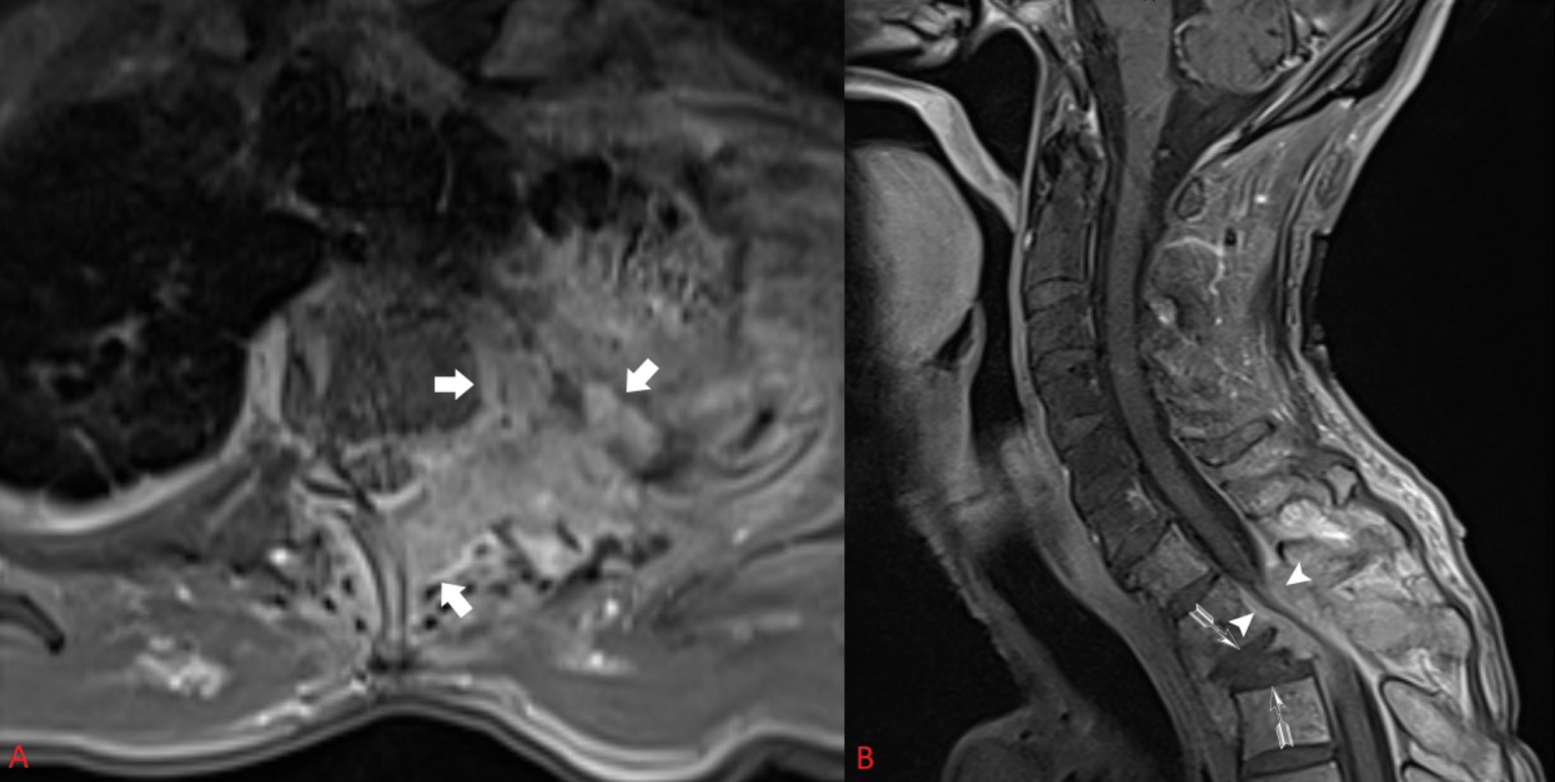
Pre-operative axial (A) and sagittal (B) post contrasted MRI demonstrate a tumor eroding through the chest wall, vertebral body, pedicle, and posterior elements causing compression at the cervical-thoracic junction, kyphotic deformity, and segmental instability Tumor invasion can be seen in the T2 Vertebral Body, Chest Wall, and Posterior Elements on axial imaging (Thick Arrows). Kyphotic deformity is denoted (Feathered Arrows). Spinal Canal Compromise is also shown (Arrow Heads).

A 60-year-old male with a history of non-small-cell lung cancer treated with chemotherapy and chest radiation over four years presented in an outpatient clinic setting with three weeks of gait ataxia and significant upper thoracic back pain. Neurological examination demonstrated subtle weakness against resistance in the lower extremities, but ataxia when ambulating. He had diminished sensation to light touch over the C8 dermatome of the left arm. He demonstrated non-sustained myoclonus in the ankles bilaterally. Sample images from a magnetic resonance image (MRI) of the cervical and thoracic spine are provided in Figure 1. 

**Figure 2:**
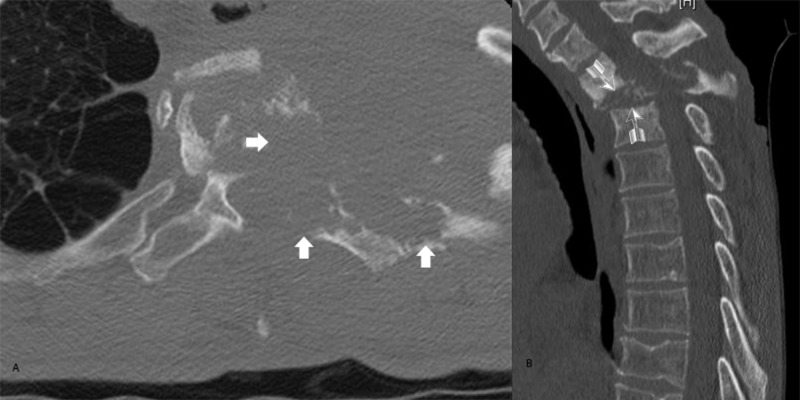
Pre-Operative (A) Axial and (B) Sagittal Bone Windowed CT Scan Thick Arrows: Tumor Invasion of bone in the vertebral body, posterior elements, and rib head. Feathered Arrows: Kyphotic Deformity

The images are notable for spinal cord compression from an epidural tumor arising from the chest wall. The patient was admitted to the hospital, and a computer-aided tomography (CT) scan of the cervical and thoracic spine demonstrated significant osseous involvement of the tumor and destruction on the left side of T1, T2, and T3 (Figure 2). Ribs, one through four, on the left, also show significant destruction from the malignancy. Given the patient’s deteriorating but functional neurological examination, surgery was offered to try and preserve his ability to ambulate for as long as possible; however, it was felt that a combined anterior and posterior decompression procedure might be greater than the patient could tolerate. The kyphotic angle of the cervical spine in relation to the thoracic spine was concerning. The patient’s pedicles measured less than 4mm in the cervical vertebra, making him a less than ideal candidate for pedicle screws at these levels. Given his malignancy and kyphotic deformity, we felt he was at high risk for failure at the top of whatever fusion construct we would create given the difficulty of pedicle screws with a small pedicle diameter.

**Figure 3:**
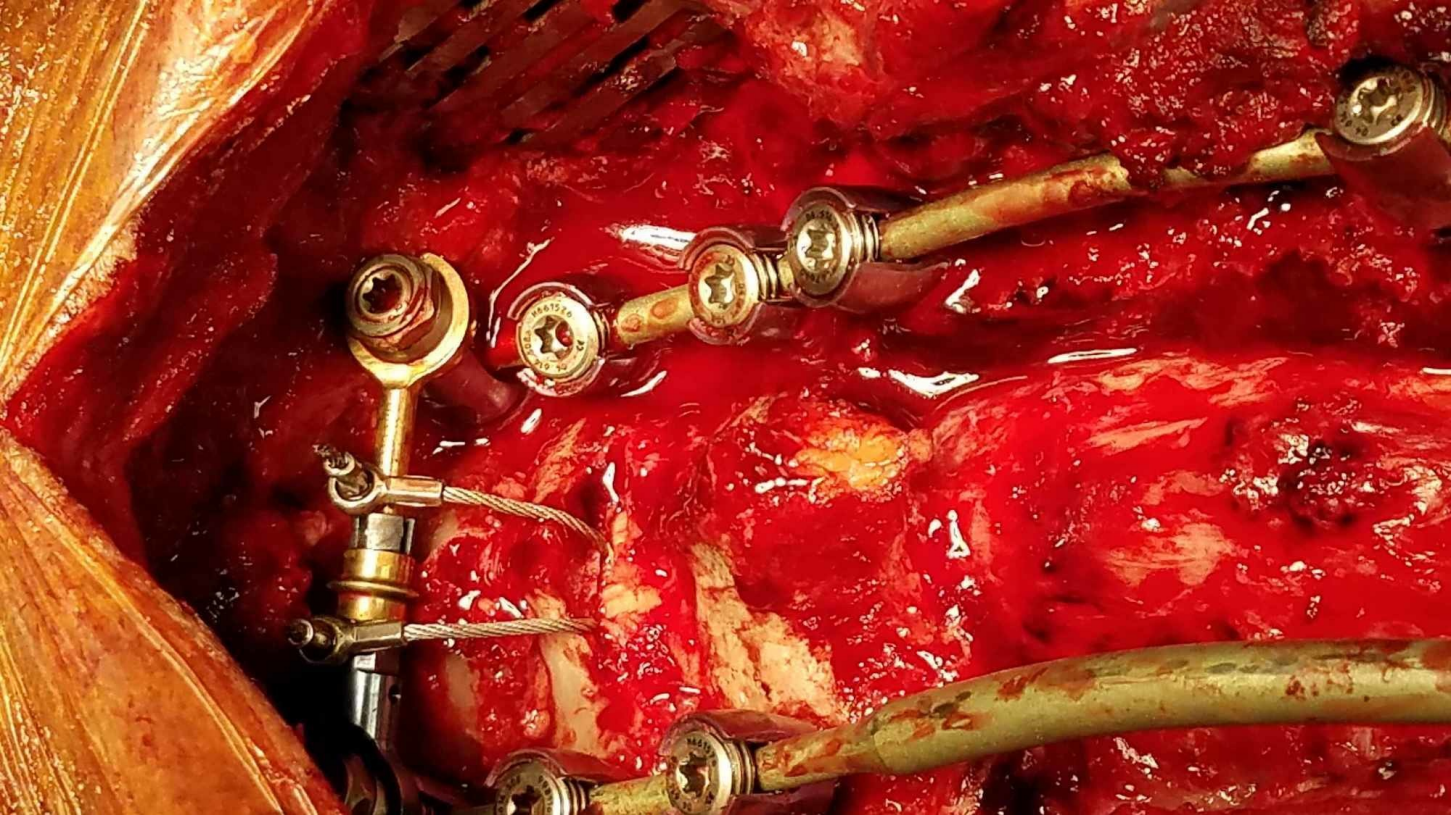
Finished construct demonstrating two sub-laminar wires threaded over the cross-link for additional stability against flexion vectors

The patient was taken to the operative suite and placed in a prone position in a Mayfield pinion head holder. The neck was slightly flexed but left essentially neutral. A midline incision was carried out from the spinous process of C4 to T6. The laminas of C5, C6, C7, T1, T2, T3, T4, and T5 were exposed. The tumor was encountered on the left side from C7 to T3. The facet joints and lateral masses were exposed laterally at C5, C6, and C7. Lateral mass screws were placed utilizing the Magerl technique at C5, C6, and C7 bilaterally. At this time O-arm navigation was brought in and utilized to place 4.5mm pedicle screws into the right T1 pedicle, right T3 pedicle, and bilateral T4 and T5 pedicles. Once the screws were placed, a decompression of the spinal cord was undertaken by removing the T1, T2, T3, and bottom portion of C7 laminae. The ligament between C4-5 and C5-6 was removed to make room for sub-laminar Atlas cables, which were passed bilaterally. A tapering rod 3.5mm to 5.5mm in diameter was bent and placed to connect the screws at each level. Then a trans-connector was placed atop the C5 lateral mass screws. The sub-laminar cables were then wrapped around the connector as an extra guard against pull out of the lateral mass screws, which would be under greater load in comparison to their thoracic counterparts, especially in the context of the patient’s kyphotic deformity. The cables were then tightened down to 35 pounds of torque. A picture of the final construct can be seen in Figure 3. 

Two 10 french round drains were left in the epidural space for the first two postoperative days to reduce the risk of an epidural hematoma. Intra-operative neurophysiological monitoring of upper extremity electromyograms (EMGs), upper and lower extremity somatosensory evoked potentials and motor evoked potentials were utilized throughout the case and demonstrated no changes. Samples were taken of the tumor, compared against previous biopsies and were consistent with a lung carcinoma that was positive for CK7, CK5/6 immunohistochemical stains and negative for TTF-1 and CK20 immunohistochemical stains.

Postoperatively the patient was placed in a cervical collar with a thoracic extension. He demonstrated continued ataxia with subtle weakness in his lower extremities against resistance. He continued to have numbness in his 4th and 5th digits of the left hand without a change in his pre-operative sensory loss pattern. After just under a week in the hospital, he was deemed a candidate for inpatient rehabilitation and was discharged to such a facility for one month’s duration.

**Figure 4:**
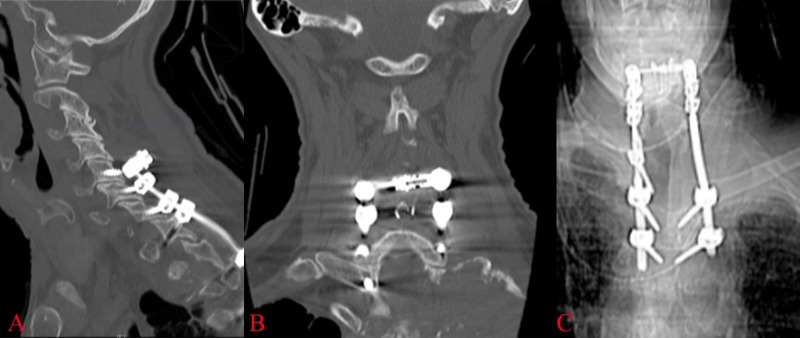
Post-fall imaging A post-fall CT scan in sagittal (A) and coronal (B) sections demonstrates hardware integrity with no evidence of pull out at the top of the construct. (C) An anterior-posterior x-ray demonstrates an intact construct in this view.

After discharge from rehab, the patient had two falls within two weeks. One of which was not in his brace and occurred when his wheeled walker became caught on a piece of furniture causing him to fall onto his right shoulder. This led him back to the emergency room where on neurological examination, he demonstrated bilateral weakness against resistance in hip flexion, knee extension, and ankle plantar and dorsiflexion. He continued to demonstrate non-sustained clonus in both ankles. Progression of the tumor had furthered cord compression at the T1, T2, and T3 levels. CT of the cervical and thoracic spine did not demonstrate any fracture or failure of the hardware. His CT scan is presented in Figure 4. The patient, with his rapidly deteriorating neurological examination, decided to pursue hospice rather than further medical, surgical, or radiation therapy options.

## Discussion

Many techniques have been described over time to aid in the stabilization of the cervical and thoracic spine from a posterior approach. Two excellent review articles describe this evolution, starting with interspinous wiring by Dr. Berthold Earnest Hadra in 1891 and evolving to cervical pedicle screw placement in 1994 by Abumi and colleagues [3,4]. Other techniques developed over time include facet wiring, interlaminar clamps, lateral mass screws and plates, lateral mass screws and rods, and sub-laminar wiring [1,2]. In traditional sub-laminar wiring, the ligament between two segments is removed or dissected such that a cable can be passed under those two laminae with a bone graft secured between the two segments. Sub-laminar wires are typically utilized in this fashion at the C1-C2 level, where anatomy can often make screw placement technically challenging, and the spinal canal anterior-posterior diameter is large. Alternatively, the wire can be passed under a single lamina and then wrapped around a rigid rod extending longitudinally over multiple segments. This approach is less seen since the advent of pedicle screws.

Construct failure is a known risk with any large instrumented fusion construct, especially in patients with poor bone quality. There are a wide variety of methods that have been described to try and improve pull out strength in such situations. These techniques include cementing screws with methylmethacrylate, the use of fenestrated screws, utilizing pedicle screws over lateral mass screws, trans-laminar screws, and others. There are few options to augment instrumentation in the sub-axial spine due to the diminutive size of the lateral masses and relatively small screw lengths. Implant failure, whether clinically symptomatic or asymptomatic, is a frequent occurrence as found by Nagashima et al. in posterior long-segment cervical fusion constructs whether at the cranial or caudal end of the construct even in a non-osteoporotic bone [5]. In their paper, they found that in over 257 pedicle screws, nine laminar screws, and 233 lateral mass screws placed into the cervical spine, screw loosening occurred in 42 screws (8.4%) and screw breakage occurred in six screws (1.2%) [5].

The technique as presented here was utilized because there was concern that poor bone quality from the patient’s malignancy, a long construct, kyphotic deformity, and lateral mass screws at the top of the construct compared to pedicle screws at the bottom of the construct would cause the patient to be vulnerable to construct failure at the top of his construct. Adding a sub-laminar wire across a cross connector adds the strength of the lamina of C5 and C6 as resistance against flexion forces. A biomechanical comparison of cervical lateral mass screws versus cervical pedicle screws, modeled by Jones et al., found a mean load-to-failure of 677 N for cervical pedicle screws compared to 355 N for cervical lateral mass screws [6]. It stands to reason that in this patient, his thoracic pedicle screws are at a higher pull out strength than his cervical lateral mass screws. We believe our sub-laminar wire to resist the key force that would cause construct failure, namely, flexion.

The diameter of the spinal canal is of key concern with any sub-laminar wiring technique. There have been several reported cases of sub-laminar wiring causing myelopathy from compression of the spinal canal and at least one case of wire break and subsequent migration [7-8]. Three studies, two by Panjabi et al. and another by Berry et al., looked at the morphometric features of the human spinal canal [9-11]. In general, it was found the diameter of the canal was at its greatest at C1-C2, and at it’s lowest between C7 and T2 [9-11]. A review of CT and MRI is critical to know if enough room exists at any level that you plan to incorporate sub-laminar wiring.

## Conclusions

A case of a 60-year-old male with metastatic carcinoma in the cervical-thoracic junction was presented, and the cancer's effect on this patient's neurological function and the biomechanical stability of his spine was discussed. Many techniques exist to stabilize this region and were reviewed. We emphasize that an understanding of anatomy is important in utilizing any stabilization technique, but especially canal diameter in the case of sub-laminar wiring. A novel technique of sub-laminar wiring to a cross-link to provide additional resistance to flexion force vectors can be used as an adjuvant in cervical-thoracic fusion constructs where the top of the construct is at high risk of pull out and failure to stabilize the spine.
